# Airway Microbiota as a Modulator of Lung Cancer

**DOI:** 10.3390/ijms21093044

**Published:** 2020-04-26

**Authors:** Taichiro Goto

**Affiliations:** Lung Cancer and Respiratory Disease Center, Yamanashi Central Hospital, Kofu, Yamanashi 4008506, Japan; taichiro@1997.jukuin.keio.ac.jp; Tel.: +81-55-253-7111

**Keywords:** microbiome, lung cancer, oncogenesis, inflammation

## Abstract

Recent research on cancer-associated microbial communities has elucidated the interplay between bacteria, immune cells, and tumor cells; the bacterial pathways involved in the induction of carcinogenesis; and their clinical significance. Although accumulating evidence shows that a dysbiotic condition is associated with lung carcinogenesis, the underlying mechanisms remain unclear. Microorganisms possibly trigger tumor initiation and progression, presumably via the production of bacterial toxins and other pro-inflammatory factors. The purpose of this review is to discuss the basic role of the airway microbiome in carcinogenesis and the underlying molecular mechanisms, with the aim of developing anticancer strategies involving the airway microbiota. In addition, the mechanisms via which the microbiome acts as a modulator of immunotherapies in lung cancer are summarized.

## 1. Introduction

The human microbiome consists of more than 1000 species of bacteria inhabiting the human body, including the skin, oral cavity, nasal cavity, stomach, small intestine, large intestine, urinary tract, and vagina (hundreds of trillions of bacteria in number) [1]. Among them, the intestinal microbiome contains approximately 40 trillion bacteria, which exceeds the total number of human cells, and plays important roles in nutrient and energy consumption of the host. The human host and the intestinal microbiome are in a symbiotic relationship, which is mutually beneficial for maintaining homeostasis [2]. Intestinal bacteria metabolize substances that cannot usually be metabolized by the host to produce energy for self-maintenance, while the host uses these metabolites for its life activities. These bacteria also protect the host from invading foreign substances and pathogenic microorganisms [3]. Loss of this homeostasis leads to the development of various diseases, including cancer [4]. Currently, the roles of the intestinal and other microbiomes in malignant tumors are being actively investigated, and studies regarding their involvement in carcinogenesis and their application in cancer treatment and prevention are underway [5]. In particular, the intestinal microbiome has been shown to modify antitumor immune activity and play an important role in regulating response and resistance to immunotherapy in malignant tumors [6].

The microbiome has been relatively well-studied in the context of obesity, inflammatory bowel disease, and arthritis. In oncology, the relationship between colorectal cancer (CRC) and certain microbiomes has been studied extensively [7,8]. Some studies have reported that microbes are involved in the malignant transformation of cells in the mucosa. In particular, the higher abundance of *Fusobacterium nucleatum*, a periodontal pathogenic bacterium colonizing the oral cavity, in the vicinity of tumors than around normal tissues in patients with CRC has prompted extensive research regarding their carcinogenesis-promoting property [7,8,9]. Studies have shown that the FadA adhesion protein complex (FadAc) expressed on the cell surface of *F. nucleatum* binds to the cell adhesion factor and E-cadherin on colonic epithelial cells to activate the β-catenin signaling pathway, thereby promoting cell proliferation [10]. In addition, *F. nucleatum* has also been shown to suppress antitumor immunity. Evidence suggests that *F. nucleatum* directly binds to T cell immunoreceptor with Ig and ITIM domains (TIGIT), an inhibitory receptor on human natural killer (NK) cells, via FAP2, thereby suppressing antitumor immunity and promoting development of CRC [11]. Higher *F. nucleatum* load has been associated with poor prognosis of CRC, suggesting the utility of this bacterium in predicting the progression and prognosis of this disease, as well as for developing strategies for its prevention and treatment [12].

Recent studies have revealed the presence of the microbiome in the lower respiratory tract; however, its association with the development and metastasis of lung cancer remains unclear. At the same time, advancements in gene analysis techniques have enabled analysis of the lower airway microbiome using 16S ribosomal RNA (rRNA) gene sequencing and metagenomic analysis, and the microbiome populations that may be involved in the development of lung cancer have been identified [3]. These microbiomes may potentially act as novel diagnostic and therapeutic biomarkers, which may facilitate the development of personalized medicine [13]. This review outlines the current knowledge regarding the role of the lower airway microbiome in carcinogenesis.

## 2. Microbiomes in the Lung and Bronchi

The culturing of intestinal bacteria using anaerobic techniques in the 1950s marked the beginning of microbiome research. At that time, when bacteria represented the main target of culture-based testing, the lower respiratory tract of healthy individuals was considered sterile in the presence of a normal immune system. As approximately 70% of the bacteria present in the human body cannot be detected using classical culture methods [14], the determination of host–microbial interactions in the lung was challenging. It was not until advancements in molecular biological techniques enabled the development of non-culture-dependent research methods in the 1980s that studies on the lower airway microbiome were initiated.

**Figure 1:**
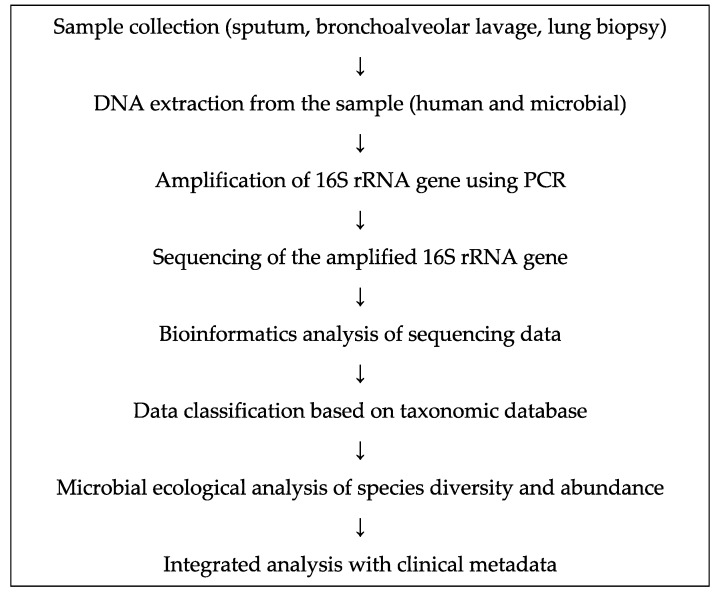
Flowchart for bacterial analysis of respiratory samples. PCR, polymerase chain reaction.

The recent development of 16S rRNA gene sequencing and metagenomic analysis has led to the identification of bacteria that cannot be detected using culture-based methods. All bacteria harbor the 16S rRNA gene, which exhibits a high level of homogeneity at the species level. As bacteria can be identified at the species and genus levels based on nucleotide sequence similarity, 16S rRNA gene sequencing is widely used at present (Figure 1).

This molecular microbial identification technique is more sensitive, less time-consuming, more efficient, and less expensive than classical culture methods. However, this method only detects DNA in a sample and does not differentiate between dead and live bacteria, a feature that distinguishes it from classical bacterial culture, which only detects live bacteria. The advent of 16S rRNA gene sequencing has led to the identification of many non-culturable bacteria, which cannot be isolated using culture-based methods. However, as pure bacterial culture was the mainstay of bacterial research at that time, the microbiome, including non-culturable bacteria, remained an unexplored area of research for a long time. Metagenomic analysis was developed in 2003 as the third bacterial detection method after culture and 16S rRNA gene sequencing. A metagenome is the sum of all the genomes of all bacteria present in a microbiome population. Therefore, analyzing a metagenome is equivalent to directly sequencing a mixture of genomes. In other words, metagenomic analysis is a method for analyzing all genetic information present in a microbiome population.

The recent advancements in sequencing technology are remarkable. Next-generation sequencers, the performance of which is several magnitudes higher than that of the sequencers used for human genome sequencing in the 1990s, have been put into practical use [15,16,17,18]. Combined with this technology, the data obtained using 16S rRNA gene sequencing and metagenomic analyses have become more comprehensible, faster, and less expensive, which has further boosted microbiome research.

Human microbiome research was initiated when the National Institutes of Health (NIH) launched the human microbiome project in 2007 to comprehensively analyze microbiomes across the human body, including the intestine, oral cavity, and skin [19,20], although the importance of the respiratory tract microbiome was not recognized at that time. The importance of microbiomes in patients with respiratory disease was first pointed out at a workshop hosted by the National Heart, Lung, and Blood Institute (NHLBI) in 2011, where it was proposed to identify and analyze microbiomes inhabiting the airway and lung, understand the role of microbiomes in lung health and disease, and develop new approaches for diagnosing and treating chronic respiratory diseases [21].

Recent progress in research on the lower airway microbiome has revealed the presence of diverse microbiomes in the lower airway of both healthy individuals and patients with respiratory diseases [22]. The fetal lung is an exception, which, along with the fetal intestine, is sterile and appears to be colonized by microbial communities only after birth. The neonatal mucosa is covered by microorganisms derived from the mother’s vagina in vaginally delivered neonates or those from the skin in babies delivered via cesarean section [23]. Although neonatal microbial communities are homogeneous throughout the body immediately after birth, unique local microbiomes start forming after several days to weeks [24]. The lung microbiome remains to be characterized in healthy neonates, but was shown to be identical to the intestinal microbiome in a longitudinal analysis of sputum samples from seven neonates with cystic fibrosis (CF) [25]. Several studies have been conducted to analyze bronchoalveolar lavage (BAL) samples from healthy adults, which revealed that the lung microbiome is composed of four phyla of bacteria, that is, Actinobacteria, Firmicutes, Bacteroides, and Proteobacteria. The phylum composition of bacteria isolated from BAL samples was almost identical to that of samples from the upper respiratory tract, including the oropharynx and nasal cavity, although some differences in composition ratio were observed. The dominant genera were *Prevotella*, *Veillonella*, *Streptococcus*, and *Pseudomonas*.

## 3. Assessment of the Role of Bacterial Microbiome Using Next-Generation Sequencing

The key consideration of any study on microbiota is the mode of studying the microbiota. This falls broadly into culture-based or culture-independent systems, or a combination of these approaches. Culture-based systems rely on the detection of specific microbes that are considered both likely to be present in a lung sample and to be important in clinical terms; detection is performed using a range of selective media and growth conditions that enable isolation of a single pure strain [26]. The earliest advancements in microbiota studies came with the development of culture-independent assays that allowed amplification of target regions directly from the DNA extracted from a clinical sample and the detection of microbial population above a set threshold [27]. Quantitative polymerase chain reaction (qPCR)-based strategies have now been developed that allow, with some calibration, an estimation of the load of species from the DNA extracted from a clinical sample. Although this approach may have fundamentally overcome many problems associated with culture bias, it still relies on a priori decision to investigate a sample for the presence of a particular species. For many years, this has been the case for virus detection in respiratory samples, where the presence of only small groups of viruses in samples, which were considered common and important, was determined. Nevertheless, these specific PCR tools constitute an important technological advancement.

Developments in the study of microbes in natural environments in terms of the methods via which the microbes in clinical samples are evaluated have heralded an important change [27]. The ribosomal RNA gene and ribosomal RNA itself are the keys to this type of assessment of cellular microbes. Ribosomal sequences—either individual genes such as the 16S rRNA gene in bacteria or the 18S rRNA gene and flanking intergenic regions in fungi—contain regions of both high sequence similarity and high sequence divergence. The regions of high sequence similarity allow for the design of primers for PCR amplification, which can be used for the determination of different taxonomic levels. For the domain bacteria, this allows primers to amplify the region spanning these sites of high conservation from potentially any bacterial cell. The regions of high sequence divergence between these primers are useful for determining the species from which an individual sequence has been derived. Bacteria possess the 70S ribosome (c.f. 80S in human), which consists of 30S and 20S subunits, and the 16S rRNA is a component of the 30S subunit. The 16S rRNA is about 1500 base pairs long and can be amplified using PCR with conserved sequences as universal primers, followed by cloning and sequencing. Currently, more than 10,000 sequences of 16S rRNA have been registered. Two bacterial isolates are considered to be related if their nucleotide sequences show ≥97% homology, and to be the same species in the case of ≥99% homology. The operational taxonomic unit (OTU) is a measure of sequence similarity, and an OTU similarity of ≥97% is considered to indicate evolutionarily identical bacterial species [28].

This key development allows the characterization of species in lower airway samples without the prior prediction of the species that might be present. A complex mix of PCR products is typically obtained using the strategy described above (sometimes called broad-range PCR). Determination of the phylogenetic identities of the species present in this complex mix forms the next step in this process. Various strategies have been developed for this purpose, which fall into two broad categories: profile-based and sequence-based [4]. The profile-based strategies allow for a relatively rapid means of assessment of the extent to which different PCR products (consequently, different species) have been amplified. These strategies are important in the context of microbiota assessment and offer a wealth of information regarding the species present. In contrast, many previous studies have combined profile-based strategies of microbiota assessment with sequence-based strategies. The original means of resolving species into single tractable units relied on the cloning of PCR products. This approach was a labor-intensive process, which meant that the number of clones studied tended to be small for any given sample. The library sizes published less than 10 years ago sometimes represented less than 100 clones. Owing to the development of next-generation sequencing (NGS), the typical number of sequences (clone equivalents) has increased by 1000-fold. However, basic information from the earlier clone libraries often matches with that of the considerably detailed libraries that are now being constructed. This can be framed as a simple question: “What is the number of a particular microbiota components present in a given sample?” Techniques such as qPCR are increasingly being used to answer this question, at least for enumeration of bacteria and panels of respiratory viruses in the respiratory system.

Traditionally, bacteria (eubacteria) are classified based on their shape, stainability, oxygen requirement, metabolic profile, and other characteristics. Currently, they are named based on taxonomic categories determined using 16S rRNA gene sequencing (Table 1). In descending order, these categories are phylum, class, order, family, genus, and species. According to the Bergey’s manual, a guide to bacterial classification and identification, 28 phyla in total can be defined using 16S rRNA gene sequencing, although up to 80 phyla can be defined if non-culturable bacteria are considered [29]. Of these, Actinobacteria, Bacteroides, Firmicutes, and Proteobacteria are the four dominant phyla inhabiting the lung of healthy individuals, the balance among which varies among organs.

## 4. Lung Microbiome in Chronic Obstructive Pulmonary Disease (COPD)

Tobacco smoke exposure can damage the epithelium and facilitate microbial entry into the host. It has also been suggested to disrupt the existing microbiome, increase the pathogenicity of microorganisms, and cause progression or exacerbation of diseases including lung cancer [30]. The lower airway microbiome has also been analyzed in respiratory diseases, such as bronchial asthma, COPD, and pulmonary fibrosis (Table 2) [31].

Lower airway samples from patients with COPD and bronchial asthma and healthy individuals were subjected to 16S rRNA gene sequencing, and the microbiome profiles of patients with respiratory disease and healthy individuals were compared [22]. Analysis of bronchoscopic brushing samples from 5 patients with COPD, 11 patients with bronchial asthma, and 8 healthy subjects showed a significant increase in the population of the members of phylum Proteobacteria, especially that of genus *Haemophilus*, in patients with COPD and adult patients with bronchial asthma, and a relative decrease in the representation of phylum Bacteroidetes, including genus *Prevotella* [22]. Initially, it was believed that the lower airway microbiome represented contamination by microbiomes in the upper airway or oral cavity. However, similar results were obtained from other studies, suggesting that contamination was unlikely [21].

Analysis of bronchoalveolar lavage fluid (BALF) samples from patients with COPD and healthy individuals showed a lower diversity of the microbiome in the patients than in the healthy individuals, where bacteria of genera *Haemophilus*, *Streptococcus*, *Prevotella*, and *Pseudomonas* were dominant, suggesting a correlation between reduced microbiome diversity and increased severity of obstructive disorder [32]. Reduced microbiome diversity has also been associated with increased severity of bronchiectasis, cystic pulmonary fibrosis, and other conditions, suggesting its association with disease activity.

Comparison of the microbiome profile in the sputum, tracheal aspirate, BALF, and bronchial mucosal biopsy samples collected from six patients with COPD showed that the microbiome profiles of BALF and bronchial mucosal biopsy samples were similar, while differences in microbiome diversity were observed in other samples, with the sputum samples showing the largest reduction in diversity [33]. An analysis of samples collected from different parts of the lungs of patients with severe COPD excised during transplantation showed obvious microbiome heterogeneity among lung lobes [34]. These findings suggest the involvement of host-specific lung microbiomes in the pathogenesis of respiratory diseases such as COPD and lung cancer via induction of local inflammatory changes.

**Table 2:** Relationship between lung microbiota and lung disease.

Respiratory Disease	Year	References	Analytical Method	Differential Findings	Sample Type	Sample Size
Chronic Obstructive Pulmonary Disease	2012	Garcha et al. [35]	16S rRNA gene sequencing	*Haemophilus influenzae*	Sputum	134
	2012	Sze et al. [36]	16S rRNA gene sequencing	*Lactobacillus*	Lung tissue	24
	2012	Pragman et al. [32]	16S rRNA gene sequencing	*Leptotrichia, Fusobacterium*	Bronchoalveolar lavage	32
	2014	Millares et al. [37]	16S rRNA gene sequencing	*Pseudomonas, Corynebacterium, Moraxella*	Sputum	16
	2014	Garcia-Nu~nez et al. [38]	16S rRNA gene sequencing	Proteobacteria, Firmicutes, Actinobacteria	Sputum	17
	2016	Lee et al. [39]	16S rRNA gene sequencing	*Prevotella, Porphyromonas, Veillonella, Fusobacterium, Streptococcus*	Sputum	8
	2017	Kim et al. [40]	16S rRNA gene sequencing	*Ochrobactrum*	Lung tissue	26
Asthma	2007	Bisgaard et al. [41]	culture	*Streptococcus pneumoniae, Moraxella catarrhalis, H. influenzae*	Hypopharyngeal	321
	2010	Hilty et al. [22]	16S rRNA gene sequencing	Proteobacteria, particularly *Haemophilus spp.*	Bronchial brushing	24
	2013	Marri et al. [42]	16S rRNA gene sequencing	Proteobacteria, *Haemophilus spp.*	Sputum	20
	2015	Teo et al. [43]	16S rRNA gene sequencing	Proteobacteria, *Streptococcus*	Nasopharyngeal	234
	2017	Durack et al. [44]	16S rRNA gene sequencing	*Haemophilus, Neisseria, Fusobacterium, Porphyromonas*	Bronchial brushing	42
	2019	Thorsen et al. [45]	16S rRNA gene sequencing	*Veillonella, Prevotella*	Airway aspirates	544
	2019	Espuela-Ortiz et al. [46]	16S rRNA gene sequencing	*Veillonella*	Saliva	57
Idiopathic Pulmonary Fibrosis	2014	Han et al. [47]	16S rRNA gene sequencing	*Staphylococcus, Streptococcus*	Bronchoalveolar lavage	55
	2017	Molyneaux et al. [48]	16S rRNA gene sequencing	*Haemophilus, Streptococcus, Neisseria, Veillonella*	Bronchoalveolar lavage	65

## 5. Lung Microbiome in Asthma

In the field of bronchial asthma, a large-scale study in children showed high indoor bacterial and fungal loads in families living on farms and an inverse correlation between microbiome diversity and asthma incidence [49]. Studies have suggested that exposure to environmental microorganisms reduces allergic diseases, such as asthma, a notion referred to as the hygiene hypothesis. Reduced exposure to pathogens during childhood can disrupt mucosal tolerance, and thereby increase the risk of autoimmune disease [50]. Many clinical studies have suggested that exposure to antibiotics during childhood increases the risk of developing asthma and allergic diseases [51].

In an investigation regarding the role of endotoxin in asthma onset, pretreatment of mouse exhibiting house dust mite (HDM)-induced asthma with endotoxin (LPS) reduced the migratory ability of dendritic cells and the expression of chemokine (C-C motif) ligand 20 (CCL 20), granulocyte macrophage colony-stimulating factor (GM-CSF), and interleukin-33 (IL-33) in airway epithelial cells, thereby improving eosinophilic inflammation and airway hyper-reactivity [52]. In this process, increase in the expression of ubiquitin-modifying enzyme A20 and the resulting reduced activation of NF-κB in airway epithelial cells resulted in inhibition of various inflammatory cascades. Endotoxin did not suppress the onset of asthma in mice with induced A20 deficiency in an airway epithelium-specific manner, suggesting an important role of A20 expression in the anti-asthma effect of endotoxin in airway epithelial cells.

The presence of a unique microbiome in the human lower respiratory tract was first reported in 2010 [22], which revealed that the lower airway microbiome in patients with asthma is characterized by the dominance of Proteobacteria (Table 2). Studies have also shown the involvement of colonization of *Haemophilus influenzae*, *Streptococcus pneumoniae*, and *Moraxella catarrhalis* in the onset of asthma in children [41,53], and that of *Haemophilus* and *Klebsiella* in the pathogenesis of Th17-mediated neutrophilic inflammation and steroid-resistant severe asthma [54]. Microbiome modification may contribute to the prevention and treatment of asthma and represents an area of further investigation.

Studies have also shown that macrolide antibiotics improve the forced expiratory volume in 1 s in patients with *Chlamydia* and *Mycoplasma* in the lower respiratory tract [55], and that antifungals were effective in patients with severe asthma sensitized to fungi [56], suggesting the effectiveness of microbiome modification by probiotics or other means for the prevention and treatment of asthma.

## 6. Lung Microbiome and Idiopathic Pulmonary Fibrosis (IPF)

Recent studies have demonstrated that the lung microbiome is also associated with the pathogenesis of IPF (Table 2) [48,57,58,59,60]. Analysis of BAL samples from patients with interstitial pneumonia, including idiopathic pulmonary fibrosis (IPF), non-specific interstitial pneumonia, and acute interstitial pneumonia, resulted in the detection of classic respiratory pathogens such as *Haemophilus influenzae* and various other pathogens that are rarely detected [61].

Molyneaux et al. prospectively enrolled 65 patients with IPF and 44 control patients (including those with COPD), and collected BALF samples for 16S rRNA gene sequencing [62]. For IPF cases, progression was defined as a 10% decrease in forced vital capacity (FVC) or death. The baseline bacterial burden, measured in terms of the 16S rRNA copy number, was higher in the IPF group than in the healthy and COPD groups. Furthermore, patients with progressing IPF at six months had higher copy numbers than those with stable IPF. When the patients in the IPF group were divided into those with high, intermediate, and low copy numbers at baseline, the high copy number group had a higher mortality rate than the other groups. Compared with that in the control group, the IPF group showed increased OTUs for *Haemophilus* sp., *Neisseria* sp., *Streptococcus sp.*, and *Vellonella* sp. In addition, the composition of these bacteria did not differ between patients with progressing IPF and those with stable IPF. These results suggest an association between bacterial burden, progression of IPF, and the potential of antimicrobial drugs for the prevention of progression, but do not support the involvement of bacterial burden in the development of IPF. Han et al. analyzed BAL samples collected from 55 patients with IPF participating in the COMET (Correlating Outcomes with biochemical Markers to Estimate Time-progression) study [47]. Progression-free survival (PFS) was defined as time to death, acute exacerbation, lung transplantation, a 10% decrease in FVC, or a 15% decrease in diffusing capacity of lung carbon monoxide (DLCO). IPF progression was associated with increased OTUs of genera *Streptococcus* and *Staphylococcus*. These results suggest the involvement of these two genera of phylum Firmicutes in the progression of IPF.

Molyneaux et al. investigated the association between microbiome variability and acute exacerbation of IPF (AE-IPF) using 16S rRNA gene sequencing of BAL samples from 20 patients with AE-IPF and 15 patients with stable IPF [48]. The composition ratios of the four main phyla, that is, Actinobacteria, Bacteroidetes, Firmicutes, and Proteobacteria, were 10%, 16%, 34%, and 32%, respectively, in the stable IPF group. In the AE-IPF group, only the ratio of phylum Proteobacteria was higher (40%) than that of the stable IPF group. The AE-IPF group showed an increase in the OTUs of *Campylobacter* sp. and *Stenotrophomonas* sp. of phylum Proteobacteria. Thus, while Han et al. suggested the involvement of *Streptococcus* and *Staphylococcus* in IPF progression [47], the results of Molyneaux et al. showed no significant change in *Streptococcus* or *Staphylococcus* load in samples from patients with AE-IPF [48]. Despite these observations, a definitive conclusion is yet to be reached. This is because of the difficulty in demonstrating the involvement of microbiomes in disease development in patients with established pulmonary fibrosis, and we may be simply looking at fluctuations in infection load or bacterial composition ratios during the course of disease progression.

Recent reports have also described the relationship between microbiomes and host response. Molyneaux and colleagues conducted a prospective study involving 60 patients with IPF and matched control patients [63]. BAL was performed and baseline whole peripheral blood samples were collected in PAX gene tubes for RNA extraction. Samples were also collected from patients with IPF for up to one year, whenever possible. The Affymetrix Human Gene 1.1 ST array was used to profile gene expression. Two gene modules were identified using network analysis. These included host defense-related genes, such as NLR family CARD domain-containing protein 4 (*NLRC4*)*,* peptidoglycan recognition protein 1 (*PGLYRPI*)*,* matrix metalloproteinase 9 (*MMP9*), and defensin alpha 4 (*DEFA4*), and genes encoding the antimicrobial peptides, secretory leukocyte peptidase inhibitor (SLPI) and cathelicidin antimicrobial peptide (CAMP). These transcription products were associated with survival. These results indicated that the lower airway microbiome plays a significant role in alveolar damage in IPF.

## 7. Microbiome and Cancer

The microbiome is attracting attention as a biomarker for cancer development as they are located in the vicinity of tumor tissues and may invade peritumoral tissues [64,65]. Certain microbiomes have been associated with increased risk of developing liver and colorectal cancers [66]. In particular, *F. nucleatum* has been associated with periodontitis and acute appendicitis, and was recently detected more abundantly around the tumor site than around normal tissue in patients with CRC [7]. In CRC, RNA sequencing revealed a marked overexpression of *F. nucleatum*-specific sequences in tumors compared with in normal tissue samples [7].

To verify the involvement of *Fusobacterium* overexpression in tumors in cancer development, quantitative PCR or real-time PCR was performed using 149 colorectal cancer tissues, 89 adjacent normal-appearing mucosal tissue, and 72 normal colonic mucosal tissue samples to detect *Fusobacterium* and gene mutations [67]. *Fusobacterium* was detected in 111 of 149 (74%) colon cancer tissue samples and was over-represented in 9% (14/149) of samples. As expected, *Fusobacterium* was detected in both tumor and normal tissues; however, the bacterial load in normal tissue was considerably lower than that in colon cancer tissue. High *Fusobacterium* load was associated with CpG island methylator phenotype (CIMP), wild-type *TP53*, human mutL homolog 1 (hMLH1) methylation, microsatellite instability (MSI), and *CHD 7/8* mutation. Of the 11 samples subjected to whole exome sequencing, two samples with high *Fusobacterium* load harbored the largest number of somatic mutations. These results indicated that increased *Fusobacterium* load is associated with specific molecular subsets of CRC and that this intestinal bacterium is involved in CRC development. In another study, tumor and normal tissues of nine patients with CRC were subjected to whole genome sequencing. Quantitative PCR and 16S rRNA gene sequencing of 95 carcinoma/normal DNA pairs showed higher *F. nucleatum* load around the tumor site than around normal tissues [9]. In another experiment, inoculation of APC^Min/+^ mice, which are prone to CRC, with *F. nucleatum* isolates from patients with inflammatory bowel disease significantly increased the number of tumors in these mice [8].

Functional studies are also in progress. One of these studies suggests that Fad-A, a virulence factor expressed on the cell surface of *F. nucleatum*, binds to the cell adhesion factors and E-cadherin on colonic epithelial cells to activate the β-catenin signaling pathway, and thereby promote cell growth, eventually enhancing carcinogenic signals and cancer formation [10]. In addition to promotion of tumor growth, *F. nucleatum* has also been shown to suppress antitumor immunity. Evidence also suggested that *F. nucleatum* binds to TIGIT, an inhibitory receptor on human NK cells, via Fap-2, thereby suppressing antitumor immunity and promoting CRC development [11].

## 8. Microbiome and Lung Cancer

Certain microbes have also been associated with an increased risk of lung cancer [1]. In prostate, lung, colorectal, and ovarian cancer screening trials with over 77,000 subjects, antibody titers for *Chlamydia pneumoniae* were significantly higher in patients with lung cancer than in the healthy volunteers [68]. Furthermore, the use of antibiotics has also been associated with an increased risk for developing lung cancer, which indicates a potential role of dysbiosis in carcinogenesis [69]. Studies have shown an association between lung cancer and *Mycobacterium tuberculosis* [70]. Overall, these studies suggested that microorganisms can contribute to lung carcinogenesis by inducing inflammation. Microbiota and its metabolites activate toll-like receptors on immune and epithelial cells, and thereby induce inflammation. Current evidence suggests that the lower airway microbiome can affect lung carcinogenesis via different mechanisms, including induction of inflammatory processes in the host, production of bacterial toxins that alter host genome stability, and release of cancer-promoting microbial metabolites [3].

Although the role of the lower airway microbiome in lung cancer development remains largely unknown (as it is a nascent field of research), several studies have been conducted to address this issue (Table 3). In a small cohort study using genomic DNA (gDNA) extracted from BALF samples obtained via bronchoscopy, the gDNA of 28 BALF samples were analyzed using PCR with primers targeting the V1–V3 regions of the bacterial 16S rRNA gene [71]. In total, 28 patients, including 20 patients with lung cancer and 8 patients with benign disease (2 with benign tumor, 3 with atelectasis, and 3 with pulmonary consolidation), were included in this study. Genera-level analysis showed a significantly higher abundance of genera *Veillonella* and *Megasphaera* in patients with lung cancer than in those with benign disease.

In a study investigating the relationship between oral microbiome and lung cancer, gDNA samples extracted from saliva were subjected to sequencing of the V3 and V6 regions of the bacterial 16S rRNA gene using the Illumina HiSeq 2000 system [72]. Genera-level analysis revealed that the abundance of genera *Capnocytophage*, *Selenomonas,* and *Veillonella* was high, whereas that of the genus *Neisseria* was low in both lung adenocarcinoma and squamous cell carcinoma (SCC) of the lung. Tsay et al. investigated the effect of host–microbial interactions in the lower respiratory tract of patients with lung cancer on known cancer signaling pathways using transcriptome analysis [73]. Brachial brushing samples collected using bronchoscopy from 39 patients with lung cancer, 36 patients with benign lung nodule, and 10 healthy volunteers were prospectively subjected to 16S rRNA gene sequencing and transcriptome analysis. Unlike healthy individuals, patients with lung cancer showed upregulation of extracellular signal-regulated kinase (ERK) and phosphoinositide 3-kinase (PI3K) signaling pathways. In the lower respiratory tract of patients with lung cancer, an increased abundance of oral bacteria (e.g., *Streptococcus* and *Veillonella*) was detected and associated with upregulation of the ERK and PI3K signaling pathways. In vitro exposure of airway epithelial cells to *Veillonella*, *Prevotella,* and *Streptococcus* resulted in upregulation of the ERK and PI3K signaling pathways, indicating that transcriptome signatures related to lung cancer etiology are associated with an increased abundance of symbiotic oral bacteria in the lower airway microbiome.

The NIH conducted a microbiome analysis of 398 patients with lung cancer [74]. DNA was extracted from paired surgical samples of lung cancer and adjacent non-tumor tissues from 121 patients who underwent surgery for early-stage lung cancer and were subjected to V3-V5 16S rRNA gene sequencing using a next-generation sequencer (Illumina MiSeq platform) and full-length 16S rRNA sequencing using the Pacific Biosciences sequencing platform. Lung cancer tissue samples were obtained from 67 patients with lung adenocarcinoma and 47 with SCC of the lung. The results showed a significantly higher abundance of *Acidovorax*, *Klebsiella*, *Rhodoferax,* and *Anaerococcus* bacteria in SCC than in adenocarcinoma. Bacteria of the genus *Acidovorax* were detected within lung cancer tumors using fluorescent in situ hybridization (FISH) in 485 patients with lung adenocarcinoma and 489 patients with SCC of the lung from the Cancer Genome Atlas program. The above results indicated that nine microbiomes containing the genus *Acidovorax* are especially abundant in patients with *TP53* mutation-positive SCC of the lung and smoking history, and can be novel biomarkers for early diagnosis of lung cancer.

The lower airway microbiome was also analyzed in BALF samples. BALF samples were collected from 91 patients with lung cancer, 29 patients with benign lung disease, and 30 healthy individuals, and were subjected to metagenomic analysis. *Bradyrhizobium japonicum* was only detected in patients with lung cancer, while the genus *Acidovorax* was detected in patients with both lung cancer and benign lung disease [75]. A microbiome diagnostic model based on age, smoking status, and 11 different microbiomes was established, which shows that the microbiome abundance in patients with lung cancer was lower than that in healthy individuals, and that microbiome-specific biomarkers are useful for diagnosing lung cancer when a lung biopsy cannot be performed.

The results of 16S rRNA gene sequencing of non-tumor tissues have also been reported [76]. Non-tumor lung tissue samples collected from 40 heavy smokers, including 10 with emphysema alone, 11 with lung cancer alone, and 19 with lung cancer and emphysema, were subjected to 16S rRNA gene sequencing. Emphysema-alone patients showed lower homogeneity of bacterial communities than patients with lung cancer (*p* = 0.006). The microbiome of patients with lung cancer was characterized by a higher abundance of phylum Proteobacteria (predominantly genera *Acinetobacter* and *Acidovorax*) and a significantly lower abundance of phylum Firmicutes (genus *Streptococcus*) and Bacteroides (genus *Prevotella*) than that of patients with emphysema. The composition of the lung microbiome in smoking patients with lung cancer differed from that in emphysema-alone patients, suggesting that changes in the lung microbiome can be a useful biomarker for lung cancer screening.

A study compared the microbiomes isolated from bronchial washing fluid (BWF) and sputum samples, focusing on the types of extracted samples and histological types of lung cancer [77]. In this study, 40 BWF and 52 sputum samples were collected and subjected to 16S rRNA gene sequencing using the Illumina HiSeq 2500 sequencer. BWF samples reflected the microbiome of lung cancer tissues better than that of sputum samples, and the microbiome composition varied depending on histological type and metastasis status [77].

Gomes et al. conducted 16S rRNA gene (V3–V6) sequencing of BALF samples and RNA sequencing of 1009 cases from the Cancer Genome Atlas to analyze the microbiome profile based on the histological type of lung cancer [78]. Microbial diversity was analyzed based on cancer subtype, smoking history, and history of airflow obstruction, among other clinical data. Members of phylum Proteobacteria were abundant in the microbiome of patients with lung cancer, and were more diverse in patients with SCC than in those with adenocarcinoma, especially in men and smokers.

**Table 3:** Review of microbiota found in patients with lung cancer.

Year	References	Analytical Method	Differential Findings	Sample Type	Sample Size
2014	Hosgood et al. [79]	16S rRNA gene sequencing	*Granulicatella, Abiotrophia,* and *Streptococcus*	Buccal and sputum samples	16
2015	Yan et al. [72]	16S rRNA gene sequencing	*Capnocytophaga, Selenomonas,* and *Veillonella*	Saliva samples	30
2016	Lee et al. [71]	16S rRNA gene sequencing	*Veillonella* and *Megasphaera*	Bronchoalveolar lavage	28
2016	Yu et al. [80]	16S rRNA gene sequencing	*Thermus* and *Ralstonia*	Lung tissues	165
2017	Cameron et al. [81]	16S rRNA gene sequencing	*Streptococcus viridans, Granulicatella adiacens, Streptococcus intermedius,* and *Mycobacterium tuberculosis*	Sputum samples	10
2018	Liu et al. [82]	16S rRNA gene sequencing	*Streptococcus*	Lung tissues and bronchoscopy samples	42
2018	Tsay et al. [73]	16S rRNA gene sequencing	*Streptococcusa* and *Veillonella*	Bronchial brushing	39
2018	Greathouse et al. [74]	16S rRNA gene sequencing	*Acidovorax, Klebsiella, Rhodoferax,* and *Anaerococcus*	Lung tissues	121
2018	Apopa et al. [83]	16S rRNA gene sequencing	*Cyanobacteria*	Lung tissues	29
2018	Liu et al. [76]	16S rRNA gene sequencing	*Streptococcus* and *Prevotella*	Lung tissues	40
2018	Yang et al. [84]	16S rRNA gene sequencing	*Sphingomonas* and *Blastomonas*	Saliva samples	247
2019	Jin et al. [75]	16S rRNA gene sequencing	*Bradyrhizobium japonicum*	Bronchoalveolar lavage	91

## 9. Possible Mechanisms of Action of the Microbiome in Lung Cancer Pathogenesis

The mechanisms via which bacteria potentially affect cancer initiation and progression were recently identified [3,4]. Smoking and gene mutations have been implicated to be involved in carcinogenesis [85]. The other factors positively related to carcinogenesis include the cells present in the cancer microenvironment and the proteins secreted from these cells, as well as the altered metabolic pathways in cancer cells leading to alterations in the proliferative capacity of the cancer cells [3]. Proteins present in intestinal bacteria, and the toxins and metabolites produced by bacteria, may also promote carcinogenesis [4].

Apopa et al. observed that CD36 may act as the connection between lung microbiota and the specific insults that contribute to lung cancer development [83]. Altered expression of CD36 in lung tissue is associated with lung cancer [86,87]. CD36 has been shown to interact with pathogen-derived ligands or toxins and is an important mediator of inflammatory pathways [88]. However, they showed that CD36 might modulate lung carcinogenesis by affecting the poly (ADP-ribose) polymerase 1 (PARP1) pathway, which is an important regulator of cell proliferation and carcinogenesis [88]. Studies have shown that CD36 regulates the internalization and processing of cyanobacteria-derived microcystin residues in the lung alveoli, increasing PARP1 expression [83]. In addition to Bacteriodetes and Proteobacteria as the most predominant phyla, Cyanobacteria (0.53%) were detected in the lung samples of patients, supporting the relevance of the mechanism described above. Furthermore, Greathouse et al. hypothesized that the interplay between smoking, TP53 mutation status, and microbiota might be relevant during smoking-driven lung carcinogenesis. Lung epithelial cells with tobacco smoke-induced mutations in TP53 are invaded by species that take advantage of the new microenvironment, suggesting that these bacteria may act as promoters of lung tumorigenesis [74]. Another study showed that ERK and PI3K signaling pathways are upregulated in vivo and in vitro after exposure of airway epithelial cells to *Veillonella*, *Prevotella*, and *Streptococcus* [73]. PI3K is a key pathway involved in the pathogenesis of non-small cell lung cancer (NSCLC), as it regulates cell proliferation and survival [89].

The mechanisms adopted by the microbiome for the modulation of immune responses in cancers were also demonstrated. Cheng et al. demonstrated the importance of commensal bacteria in maintaining immune homeostasis against cancer, revealing defective induction of lung immunity after antibiotic treatment [90]. On the other hand, Le Noci et al. observed that local antibiotic treatment reduces the implantation of experimental lung metastases and that this effect is associated with the modulation of immune response [91]. These data demonstrated that the commensal bacteria may establish a permissive environment for cancer in some circumstances.

## 10. Effect of Microbes on Cancer Immunotherapy

A small number of cancer cells in the body are usually attacked and eliminated by the antitumor immune mechanism [92]. However, this mechanism may gradually malfunction as cancer cells grow. Regarding the cause of impaired antitumor immunity, data suggest the involvement of the immune checkpoint mechanism, which suppresses immunity, and thereby allows tumors to evade immunity [92,93,94]. The immune checkpoint mechanism is necessary for the suppression of excessive immune activation, but can facilitate cancer progression in cancerous tissue, thereby adversely affecting the host [95]. Currently, immune checkpoint inhibitors (ICIs) have attracted attention. Antibodies against immunosuppressive molecules, such as programmed cell death-1 (PD-1) and its ligand (PD-L1) and cytotoxic T-lymphocyte antigen-4 (CLTA4), have been developed as ICIs [96]. However, all patients with cancer do not respond to ICI treatment. Furthermore, recent studies have suggested that some intestinal bacteria are capable of enhancing antitumor immunity and augmenting the effect of ICIs [97,98,99]. A study using a mouse model showed that *Bifidobacterium* activated dendritic cells, and thereby enhanced antitumor immunity and augmented the antitumor effect of the anti-PD-L1 antibody [97]. In another study, *Bacteroides* spp., in particular *Bacteroides thetaiotaomicron* and *Bacteroides fragilis*, augmented the effect of the anti-CTLA-4 antibody in a mouse model of CRC [98]. These results suggested that, for cases that do not respond well to ICI treatment, modification of the intestinal microbiome profile may reverse the effect of these treatments [100].

In fact, antibiotics have been shown to inhibit the effect of ICIs in patients with advanced cancer [101]. Fecal microbiota transplantation (FMT) from cancer patients responding to ICIs to sterile or antibiotic-treated mice improved the antitumor efficacy of the PD-1 blockade. These results indicated that primary resistance to ICIs is partially attributable to abnormal intestinal microbiome composition. However, the relationship between the lower airway microbiome and ICIs has not been extensively investigated and remains an area of future research. In an experimental mouse model, Le Noci et al. showed that the aerosolization of bacteria isolated from lung microbiota of antibiotic-treated mice reduced lung metastasis implantation by enhancing cancer immune response [91]. This study also showed that lung microbiota might be manipulated by antibiotic or probiotic aerosolization, and that these changes are associated with reversion of immunosuppression observed in the tumor microenvironment, favoring the immune response against cancer cells.

## 11. Conclusions

This article reviewed our current knowledge on microbiomes and carcinogenesis, focusing on the relationship between the lower airway microbiome and lung cancer. The existence of microbiota in the lower airways has been demonstrated using novel sequencing techniques, and their pathological significance may vary with the host, lung biology, and exposure to microbes. Significant evidence supports the plausibility of this association with potential therapeutic implications. Whether modulation of microbiomes can lead to cancer prevention or treatment has not yet been established. The analysis-related issues to be addressed include selection of clinical samples, DNA extraction methods, platforms for 16S rRNA gene sequencing, and improvement of analytical techniques. The role of microbiomes in carcinogenesis is currently being investigated and remains to be completely elucidated. Future large-scale studies will provide new insights regarding the microbiome-based prevention and treatment of lung cancer.

## Figures and Tables

**Table 1:** Taxonomic categories of bacteria.

*Escherichia coli* is Categorized as	Taxonomy
*Bacteria*	Domain
*Monera*	Kingdom
*Proteobacteria*	Phylum
*Gammaproteobacteria*	Class
*Enterobacteriales*	Order
*Enterobacteriaceae*	Family
*Escherichia*	Genus
*coli*	Species

The scientific names of bacteria are expressed by the two-name method: the genus name followed by the species name (e.g., *Escherichia coli*).

## Abbreviations

CRC	Colorectal cancer
NSCLC	Non-small cell lung cancer
SCC	Squamous cell carcinoma
PFS	Progression-free survival
PD-1	Programmed cell death-1
PD-1L	PD-1 ligand
CTLA-4	Cytotoxic T-lymphocyte antigen-4
ICI	Immune checkpoint inhibitor
FMT	Fecal microbiota transplantation
PI3K	Phosphoinositide 3-kinase
CIMP	CpG island methylator phenotype
IPF	Idiopathic pulmonary fibrosis
gDNA	Genomic DNA
COPD	Compulsive obstructive pulmonary disorder
TIGIT	T cell immunoreceptor with Ig and ITIM domains
NK	Natural killer
CCL20	Chemokine (C-C motif) ligand 20
GM-CSF	Granulocyte macrophage colony-stimulating factor
IL	Interleukin
NLRC4	NLR family CARD domain-containing protein 4
PGLYRP1	Peptidoglycan recognition protein 1
MMP9	Matrix metalloproteinase 9
DEFA4	Defensin alpha 4
SLPI	Secretory leukocyte peptidase inhibitor
CAMP	Cathelicidin antimicrobial peptide
hMLH1	Human mutL homolog 1
ERK	Extracellular signal-regulated kinase
PARP1	Poly (ADP-ribose) polymerase 1
